# Postherpetic Neuralgia With Sciatic Nerve Paresis Treated With Peripheral Nerve Stimulator Implantation Device

**DOI:** 10.7759/cureus.98845

**Published:** 2025-12-09

**Authors:** Daniela N Frunza, Christine LeRoy, Bijal Sheth, Nathaniel Grosu

**Affiliations:** 1 Family Medicine, St. Joseph's Regional Medical Center, Paterson, USA; 2 Family Medicine, St. Joseph's Regional Medical Center, Clifton, USA; 3 Biological Sciences, Rutgers University, New Brunswick, USA

**Keywords:** atypical shingles presentation, chronic pain management, herpes zoster paresis, herpes zoster sciatica, peripheral nerve stimulator, postherpetic neuralgia, segmental paralysis associated with herpes zoster

## Abstract

Herpes zoster, commonly known as shingles, is caused by reactivation of the latent varicella zoster virus in the sensory ganglia. It typically presents as painful grouped vesicles confined to one or two dermatomes. Most lesions resolve within two to four weeks; however, a subset of patients go on to develop postherpetic neuralgia in the area of the original zoster rash, characterized by allodynia and hyperalgesia lasting months or even years. The risk increases with age, immunosuppression, and underlying conditions such as cancer, kidney or liver disease, chronic lung disorders, etc. Although the use of oral antiviral medications during the early stages may reduce the severity and duration of acute herpes zoster, their effect on preventing postherpetic neuralgia remains limited. In this article, we report the case of a 75-year-old female with severe postherpetic neuralgia involving the left sciatic nerve distribution, with resulting motor paresis. After an extensive trial of various oral medication regimens and a lumbar epidural injection, which provided short-term relief, the patient underwent peripheral nerve stimulator (PNS) implantation, with eventual favorable response. Our case report highlights the potential role of using PNS to treat severe postherpetic neuralgia refractory to mainstay conservative treatment modalities.

## Introduction

Postherpetic neuralgia is a sequela following recovery from an acute outbreak of herpes zoster vesicular eruption. It is characterized by allodynia and hyperalgesia, most commonly between dermatomes T1 and L2. The pain associated with postherpetic neuralgia can severely affect a person’s quality of life, and in extreme cases, can be debilitating [1]. Though herpes zoster generally affects the head, neck, and trunk, herpes zoster radiculopathy of the sacral segments can be particularly challenging to diagnose and treat, as it can overlap with other spinal pathologies. Currently, first-line management for this condition focuses on maximizing pharmacotherapy with tricyclic antidepressants, gabapentinoids, SNRIs, lidocaine patches, capsaicin topicals, and, if severe enough, opioids. Unfortunately, a subset of patients responds very poorly to the above-mentioned strategies. Several interventions have been documented in the literature, including subcutaneous Botulinum Toxin A injections, transcutaneous electrical nerve stimulation, local triamcinolone injections, intrathecal injections with methylprednisolone, stellate ganglion blocks, dorsal root ganglia destruction, pulsed radiofrequency, spinal cord stimulation, paravertebral blocks, and peripheral nerve stimulation [2]. Currently, there is no uniform consensus regarding the single best interventional treatment. Our case report illustrates the use of a peripheral nerve stimulation (PNS) implantation device to alleviate severe postherpetic neuralgia of the sciatic nerve with motor paresis.

## Case presentation

**Figure 1:**
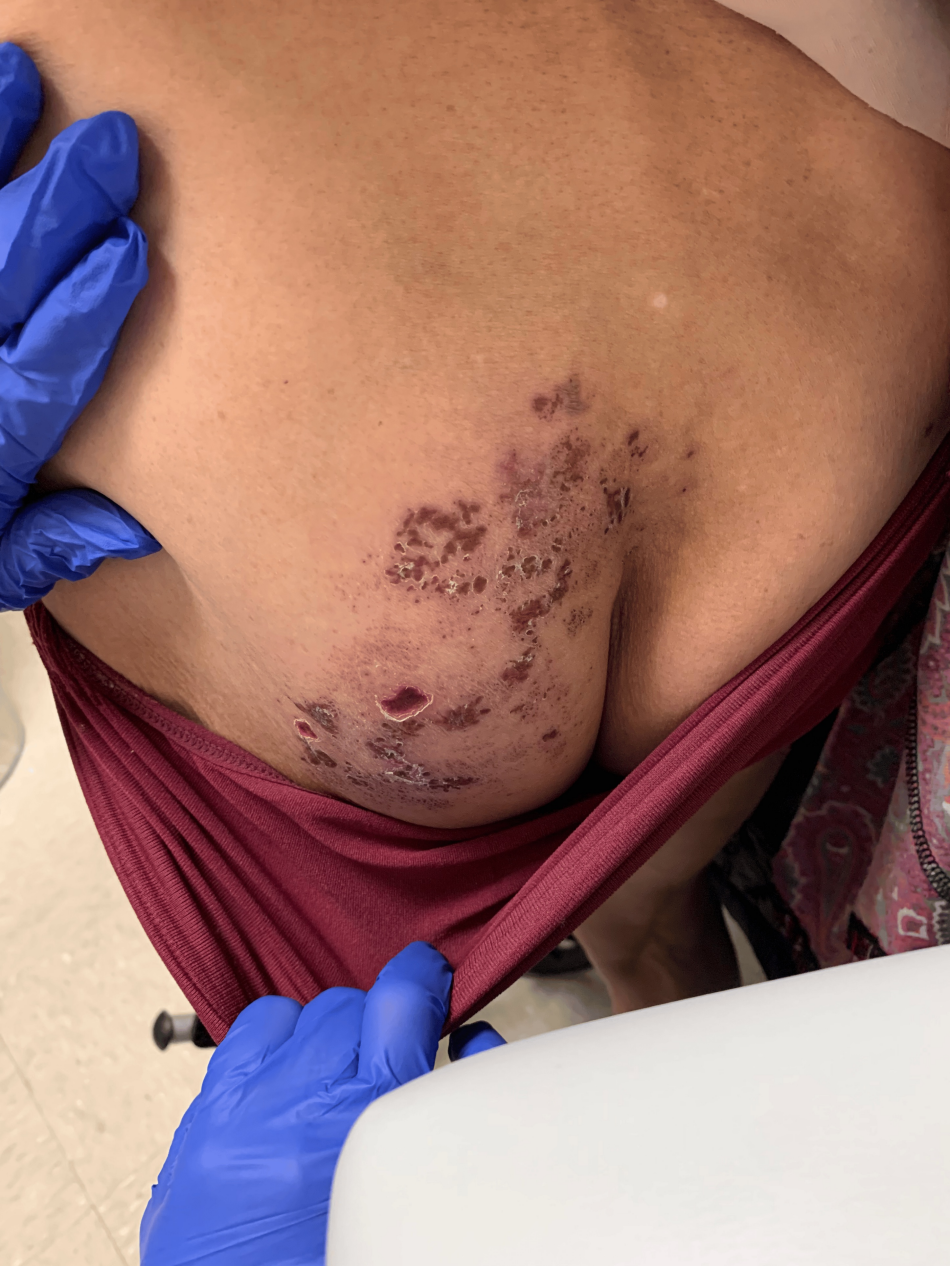
Early onset herpes zoster with multiple dried, crusted over vesicular lesions with underlying erythema extending from the left buttock downwards in an l5/s1 dermatomal distribution.

**Figure 2:**
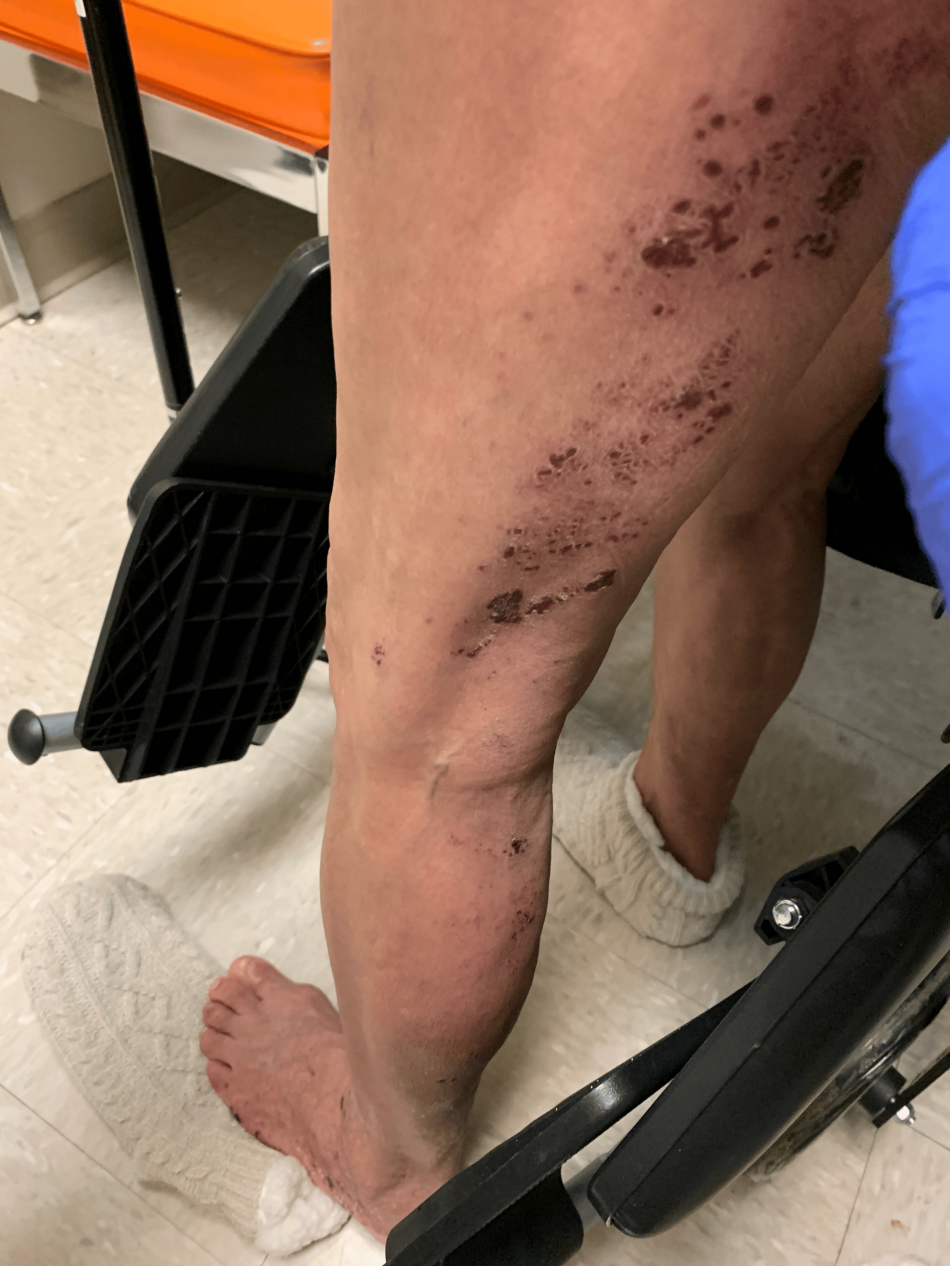
Early onset herpes zoster with multiple dried, crusted over vesicular lesions with underlying erythema extending down the left leg and the dorsal foot in an L5/S1 dermatomal distribution.

Our patient is a 75-year-old female who came to the clinic for evaluation of a red, itchy, painful rash extending from the left buttock posteriorly down the left leg and to the dorsal foot. The pain was described as shock-like, rated 10/10 in intensity, and caused her to ambulate with an antalgic gait. On initial physical exam, there were multiple dried, crusted over vesicular lesions with underlying erythema extending down the left leg along the L5/S1 dermatome (Figures 1-2). She was initially treated with a course of valacyclovir and nonsteroidal anti-inflammatory drugs (NSAIDs) for shingles. After several weeks, the rash resolved, but dysesthesia and allodynia in the distribution of the original zoster rash progressively worsened. Physical exam was significant for allodynia to light touch over the plantar aspect of the left foot and posterior aspect of the left calf. These compromised the patient’s ambulatory function to the extent that she needed to use a rolling walker and a wheelchair. She required daily assistance with eating, bathing, toileting, and dressing.

The patient underwent an extensive trial of different medication regimens over the course of several months, including antivirals, NSAIDs, topicals, tricyclic antidepressants, gabapentinoids, and opioids, which provided minimal pain relief. She was on a daily regimen of pregabalin 150 mg Q8H, acetaminophen 975 mg Q8H, and nortriptyline 25 mg QHS for several months. She also underwent transforaminal epidural steroid injections at the L4-L5 level and trigger point injections of the thoracic area, which proved moderately effective for two to three weeks at a time, after which the pain returned to its baseline. At subsequent office visits, the patient’s pain score by visual analog scale remained a constant 10/10 despite the above measures.

After four months of poor response to conventional pharmacologic modalities and debilitating pain, the medical team opted to proceed with a PNS implantation device along the left sciatic nerve. The patient tolerated the procedure well, and there were no postoperative complications.

The patient used the PNS device round the clock during the first 2 months, after which she tried to slowly de-escalate. She was able to taper off pregabalin over the course of 3 months. At the 3-month postoperative follow-up, the patient reported significant improvement and used only acetaminophen on an as-needed basis. She was able to gradually resume her activities of daily living, including feeding, bathing, walking, dressing, etc. At the last follow-up visit, roughly 4 months post-implantation device, the patient reported discontinuing PNS usage during nighttime, with as-needed use during the daytime. She expressed contentment with the gradual return of her sleep and functional status. She did not endorse any adverse effects of the PNS device.

## Discussion

Diagnosing herpes zoster radiculopathy of the lower extremities can be challenging. Though shingles generally affects the head, neck, and trunk, a skin rash involving the lower extremities can be easily mistaken for various other vesicular rashes. Moreso, motor neuron involvement, such as weakness and paresis, can mimic other spinal pathologies, such as sciatica, and in the absence of the classic zoster rash, can make the diagnosis challenging. The majority of cases of zoster motor paralysis affect the cranial nerves, such as the facial nerve. Upper limb motor nerves are more frequently affected than their counterpart lower limb motor nerves. In most cases, motor paralysis tends to occur in the same segmental distribution as the initial cutaneous zoster rash [3,4].

It is presumed that inflammation of the dorsal root ganglion extends to the nearby spinal nerve or ventral root, thereby resulting in zoster motor paresis. The literature highlights numerous case reports of such occurrences. One case report described a 75-year-old female with herpes zoster involving the sciatic nerve developing after sequential hip joint surgery [5]. Other reports have been issued on herpes zoster occurring within surgical wounds and after trauma [6]. In these atypical cases, it is presumed that direct irritation of the nearby dorsal root ganglia most likely caused sufficient stress to reactivate VZV. A more unusual disease course includes Parsonage Turner Syndrome, whereby viral insults cause unilateral brachial plexopathy [7]. Our case involves a 75-year-old female with shingles developing in the L5/S1 distribution, later complicated by motor involvement of the sciatic nerve.

Current first-line treatments for postherpetic neuralgia include medication management with tricyclic antidepressants, gabapentinoids, and capsaicin topicals, with escalation to opioids as second-line therapies. Clinicians may consider more invasive interventions when there is persistent debility despite conventional treatments, such as subcutaneous Botulinum Toxin A injections, stellate ganglion blocks, dorsal root ganglion destruction, paravertebral blocks, and PNS, among others [8]. One case report of a 75-year-old man with postherpetic neuralgia in the right L5 distribution was treated successfully with a fluoroscopically guided L5 nerve root block with Depo-Medrol and Lidocaine. It is hypothesized that injecting a steroid agent into the epidural space or along the spinal root will decrease inflammation, thereby reducing pain and weakness [9, 10].

In contrast to spinal injections, spinal cord stimulation (SCS) and PNS deliver electrical stimuli to the spinal cord or peripheral nerves, changing how pain perception reaches the brain. PNS involves implanting subcutaneous electrodes along the target peripheral nerve to provide continuous stimulation-induced analgesia by an external generator. PNS technology changes the frequency and waveforms so that patients receive customized analgesia in the affected region. The concept is comparable to the techniques of acupuncture and TENS.

PNS may be implanted along the peripheral nerves of the upper and lower extremities, as well as the abdomen, trunk, back, and pelvis. A brief review of the literature shows PNS being used to treat injuries to the occipital, ilioinguinal, supraorbital, and trigeminal nerves. With regards to postherpetic neuralgia, PNS has been used to target the supraorbital and thoracic regions, with promising outcomes.

The safety and efficacy of implanted neuromodulation devices have seen general favorability among clinicians and patients. Safety limitations to consider include device-related complications such as infections, bleeding, neural injuries, lead migrations and fractures, device malfunctions, among others. Newer devices on the market today have added to the growing popularity of PNS to treat various neuropathies [11, 12].

## Conclusions

We present a case report describing the management of severe postherpetic neuralgia involving the sciatic nerve using subcutaneous PNS. Our patient endorsed marked improvement of baseline pain with PNS after inadequate pain relief from mainstay oral medications. It is important to note that the disease course of postherpetic neuralgia may resolve spontaneously within weeks to months, making it more difficult to attribute the resolution of symptoms to PNS. Despite limited evidence regarding using this intervention for managing refractory postherpetic neuralgia, PNS offers great potential as a minimally invasive, low-risk procedure for those who have failed to obtain pain relief from mainstay conservative treatments.
